# Monitoring Public Health Through a Comprehensive Primary Care Database in the Netherlands: Overview of the Nivel Syndromic Surveillance System

**DOI:** 10.2196/58767

**Published:** 2025-03-12

**Authors:** Christos Baliatsas, Jojanneke van Summeren, Sander van Beusekom, Amy Matser, Mariette Hooiveld

**Affiliations:** 1 Nivel – Netherlands Institute for Health Services Research, Otterstraat 118, Utrecht, 3513 CR, The Netherlands, 31 629034652; 2 Infectious Diseases, Amsterdam UMC, location University of Amsterdam, Amsterdam, The Netherlands; 3 Infectious Diseases, Amsterdam Institute for Infection and Immunity, Amsterdam, The Netherlands; 4 Amsterdam Public Health, Amsterdam, The Netherlands

**Keywords:** surveillance, monitoring, general practice, public health

## Abstract

**Background:**

Syndromic surveillance systems are crucial for the monitoring of population health and the early detection of emerging health problems. Internationally, there are numerous established systems reporting on different types of data. In the Netherlands, the Nivel syndromic surveillance system provides real-time monitoring on all diseases and symptoms presented in general practice.

**Objective:**

The present article introduces the national syndromic surveillance system in primary care, emphasizing its role in providing real-time information on infectious diseases and various health problems at the population level, in the Netherlands. In addition, we report on the central role of the participating general practices in data provision, and discuss the applicability of the syndromic surveillance data in different contexts of public health research.

**Methods:**

The Nivel syndromic surveillance system is part of the Nivel Primary Care Database (Nivel-PCD) that collects routinely recorded data from electronic health records of about 10% of the Dutch population, on the basis of approximately 500 practices. This translates to approximately 1.9 million citizens. Since 2010, the surveillance system relies on representative, pseudonymized data collected on a weekly basis from a subset of about 400 practices in the Nivel-PCD, for the entire practice population. Health problems are registered according to the International Classification of Primary Care, applied in all general practices in the Netherlands. Prevalence rates are recalculated and reported every week in the form of figures, also stratified by age, sex, and region. Weekly rates are defined as the number of people that consulted the general practitioner in a certain week for a specific health problem, divided by the total number of registered individuals in the practice.

**Results:**

While utilizing data from general practitioners’ electronic health records, the system allows for the timely monitoring and identification of symptom and disease patterns and trends, not only among individuals who seek primary health care, but the entire registered population. Besides their use in disease monitoring, syndromic surveillance data are useful in various public health research contexts, such as environmental health and disaster research.

**Conclusions:**

The Nivel syndromic surveillance system serves as a valuable tool for health monitoring and research, offering valuable insights into public health.

## Introduction

Syndromic surveillance comprises the systematic, automated collection, analysis, and evaluation of real-time health data for the early monitoring and detection of changes in health indicators [1-3]. Data can be routinely collected from different sources, such as primary (general practitioners; GPs) or secondary care (emergency department visits) [4-6], depending on the health system structure. Other information sources can also be considered, such as helpline calls, medication sales, and internet searches, although they are less commonly used [4,6,7]. The broad scope of syndromic surveillance and the rapid data collection procedures can provide great flexibility for timely assessment of different health outcomes on a large population scale [6-8]. Many countries, also in Europe, have successfully incorporated syndromic surveillance into national surveillance systems [5,9-12], the role of which was crucial during the COVID-19 pandemic, despite inherent limitations [7,8,13,14].

There are well-established national networks that make use of primary care data under strict privacy regulations, for health (services) research and monitoring, for instance in the United Kingdom and Belgium [5,15,16], offering important insights into population health. In the United States, the integration of routine anonymized primary care data is more limited, primarily due to regulatory restrictions, with greater reliance on secondary health care data [17]. In the Netherlands, the Nivel (Netherlands Institute for Health Services Research) Primary Care Database (Nivel-PCD; formerly known as the Netherlands Information Network of General Practice) [17,18] was developed in the early 1990s.

Within this infrastructure, the Nivel syndromic surveillance system provides real-time information on all diseases and symptoms presented in general practice, utilizing data from a large network of GPs. Despite its important role nationwide, there is currently relatively limited international visibility of this infrastructure as well as its applicability in different research contexts. The present paper has the following main objectives: (1) to provide an overview of the Nivel syndromic surveillance system, detailing its methodology; (2) to discuss the important role of the participating general practices in providing data; and (3) to outline the diverse applications of syndromic surveillance data in public health research, highlighting its utility in identifying emerging health threats and supporting research on the health impacts of various exposures.

## Methods

### Standardized Registration in Dutch General Practices

In the Netherlands, GPs play a vital role as the first point of contact for health care services, acting as gatekeepers for specialized secondary care. Almost every individual is registered at a general practice located in the broader vicinity of their residence. GPs keep detailed electronic health records (EHRs) of consultations, diagnoses, prescribed medications, and referrals. Dutch GPs adhere to the NHG (Dutch College of General Practitioners) guidelines, specifically the ADEPD (adequate record keeping in electronic patient records) guideline, which ensures consistency, reproducibility, and standardization in medical registries, on the basis of a well-structured classification system [19,20]. More specifically, health problems and symptoms documented in general practice adhere to the International Classification of Primary Care (ICPC) [21,22], while medication prescriptions are classified according to the anatomical therapeutic chemical (ATC) classification system [23]. This EHR registration system ensures that information on both consulting and nonconsulting populations is available, which is of high importance for epidemiological research and monitoring. The data recorded by general practices, therefore, provide a comprehensive view of the health characteristics of not only primary care patients but also the broader population.

### Nivel-PCD: A Large, Nationally Representative Primary Care Database on Routinely Recorded Data

The Nivel is an independent, nonprofit, research institute that is part of the Dutch Ministry of Health’s knowledge infrastructure. The Nivel-PCD [18] is a large research infrastructure based on real world data extracted from routinely kept EHRs of GPs; primary care out-of-hours services; and paramedical care physicians such as physiotherapists, exercise therapists, and dieticians. There are approximately 500 general practices across the Netherlands that participate yearly in the Nivel-PCD [18], while the total number of general practices in the country is nearly 5000, comprising about 12,000 GPs [24].

### Data Collection and Processing

The Nivel gathers data from approximately 10% of the Dutch population (~1.9 million citizens) registered in general practices and about two-thirds of the population considering the use of out-of-hours services. Additionally, data are collected from sentinel general practices (Nivel Peilstations), where a subset of patients with influenza-like illness or other acute respiratory infections provide samples for laboratory testing. These samples are analyzed by the National Institute for Public Health and the Environment (RIVM) to identify the presence of influenza and other respiratory viruses.

For the Nivel syndromic surveillance system, EHR data are collected, extracted, and delivered by the EHR software providers used by the general practices. The system relies on pseudonymized data, available weekly since 2010, on patients consulting for certain health problems and conditions recorded in approximately 400 general practices from all over the country that provide data on a weekly basis. Pseudonymization refers to assigning nonidentifiable identification numbers to the registered practice population. The focus lies on observing and documenting trends in symptoms and diseases, presenting a timely and realistic view of health problems presented in primary care, including influenza-like illness, COVID-19, and other infectious and noninfectious diseases in the Dutch population.

The Nivel-PCD adheres to Dutch data protection regulations and laws for health data use in epidemiological research (Dutch Civil Law, Article 7:458). Medical and personal information are separated with Trusted Third Party support, preventing access to identifiable patient details. Participating health care providers can withdraw from the Nivel-PCD anytime. An opt-out system is available for patients who do not want their data to be used. The Nivel-PCD is ISO27001 certified.

### Ethical Considerations

The use of EHRs for research purposes is allowed under certain conditions. When these conditions are fulfilled, neither obtaining informed consent from patients nor approval by a medical ethics committee is obligatory for this type of observational studies containing no directly identifiable data (art. 24 GDPR Implementation Act jo art. 9.2 sub j GDPR).

## Results

### Analysis and Reporting

**Figure 1.**
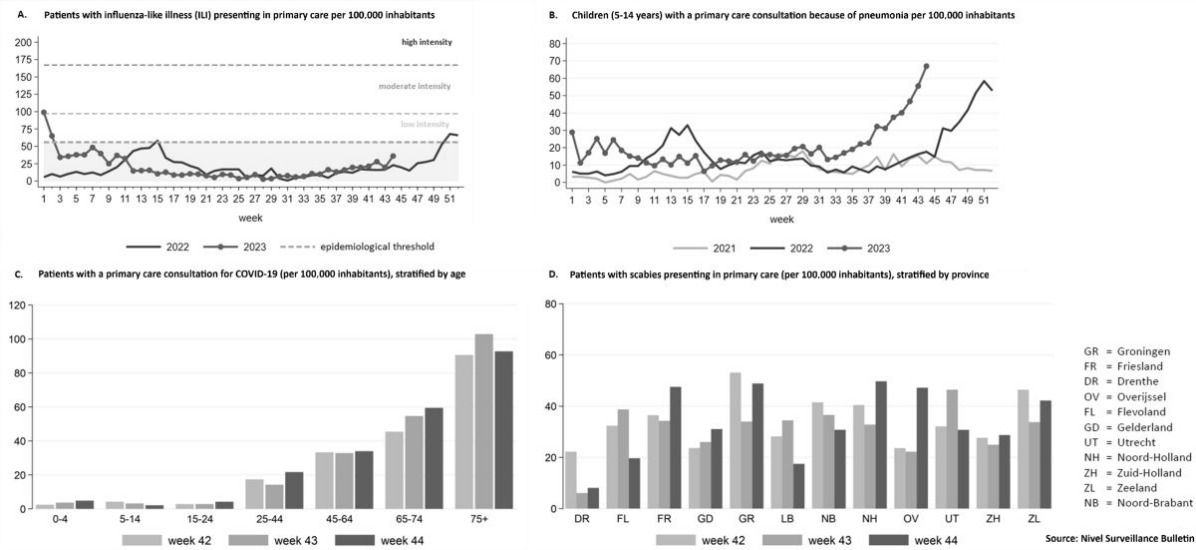
Example of four data representations from the Nivel Surveillance Bulletin as of week 44, 2023: ****A**. **Number of patients with influenza-like illness presenting in primary care per week; ****B**. **Weekly number of patients in the age group 5‐14 years with a primary care consultation because of pneumonia; ****C**. **Weekly number of patients with a primary care consultation because of COVID-19, by age group; ****D**. **Number of patients with scabies presenting in primary care per week, by province. All numbers are expressed per 100,000 inhabitants.

**Figure 2.**
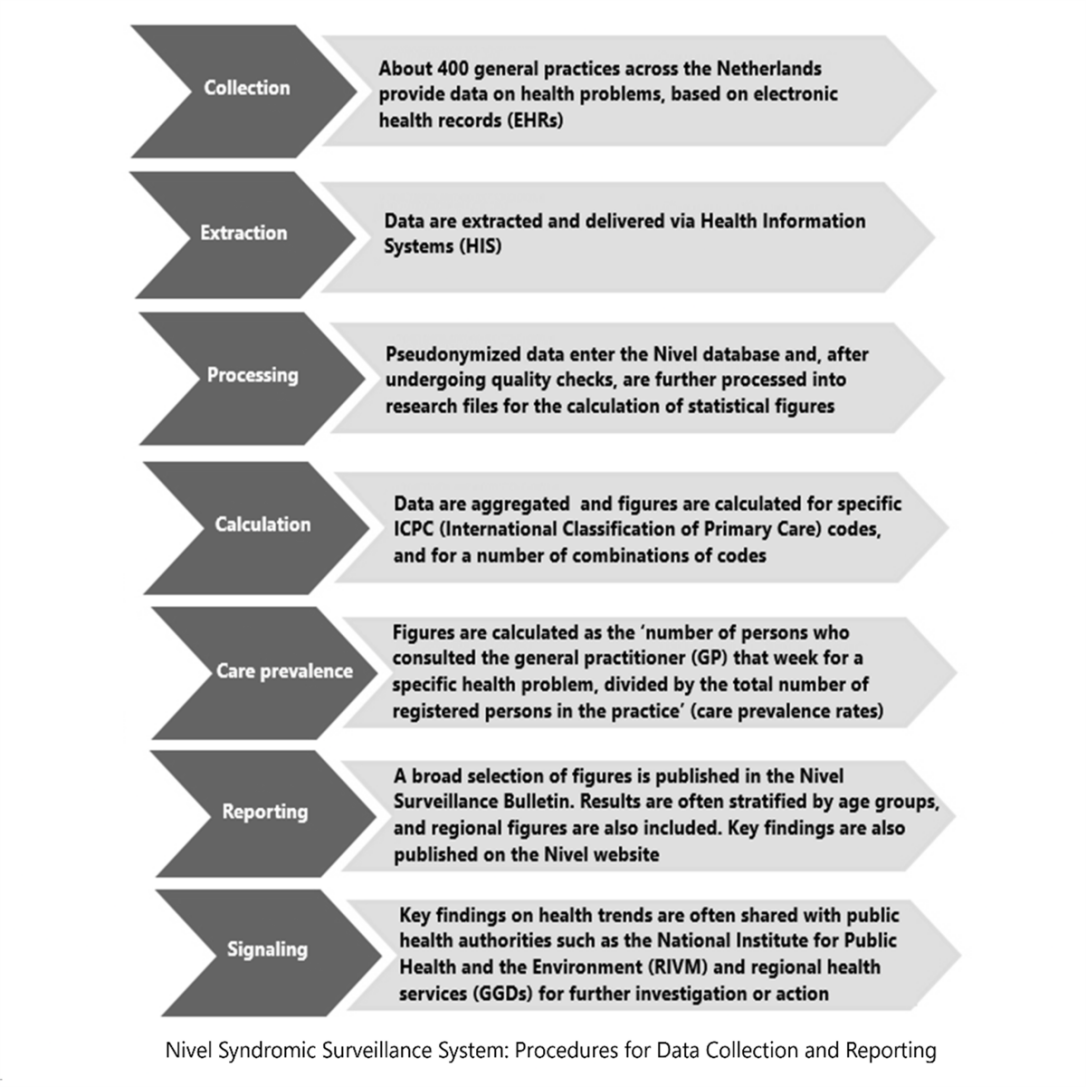
Overview of the main steps and tasks involved in data collection and report of the Nivel syndromic surveillance system.

Rates are generated for specific diagnostic codes and for a number of ICPC code combinations (eg, R74, R75, R77, R78, R80, and R83.03 for the cluster “acute respiratory infections excluding pneumonia”); the rates are defined as the number of people who have consulted the GP in that week for a certain health problem, divided by the total number of registered people in the practice. The weekly care prevalence rates are also stratified by age and sex, and by province or public health regions where the local Public Health Services operate, to monitor regional differences. The figures obtained in the previous week are recalculated weekly with any additional data provided, in order to be as up-to-date and complete as possible. The key findings are summarized weekly and published in the Nivel Surveillance Bulletin, accessible on the Nivel website [25] (see example in Figure 1). The report includes selected figures, often categorized by age groups and regions, with additional figures available upon request. An example is the notable increase in the number of children with pneumonia, reported by the Nivel syndromic surveillance system (Figure 1). This early warning initiated further investigation by the Nivel and National Institute for Public Health and the Environment (RIVM) to determine the cause of this increase. Since the start of the COVID-19 pandemic in early 2020, the system has been monitoring cases in primary care. First, the system used the free text describing the disease episode and manually categorized patients clinically diagnosed with COVID-19 or COVID-19-like illness. After the introduction of the ICPC subcode R83.03 in November 2020, the monitoring continued using the registration of this diagnosis code. Figure 2 summarizes the main steps in data collection, analysis and reporting of the Nivel syndromic surveillance system.

### Diverse Applications of Syndromic Surveillance in Different Population Research Contexts

Syndromic surveillance also extends beyond monitoring health problems reported in the Surveillance bulletin. Its infrastructure has been used for studies on different topics and contexts, for instance on the physical and psychosocial impact of the COVID-19 pandemic [26,27] as well as to monitor the presentation of symptoms in primary care in relation to environmental exposures and heat waves [28,29]. The comprehensive patient registry in the Nivel-PCD enables continuous tracking of patients over time, linked with additional sources such as socioeconomic data from Statistics Netherlands (CBS), geographic information, and environmental exposure data. Integration occurs across aggregated levels using pseudonyms to ensure patient privacy.

## Discussion

### Study Findings and Comparisons With Previous Works

The Nivel syndromic surveillance system provides large scale monitoring of diseases and health symptoms, based on representative population health data routinely registered in general practice in the Netherlands, on the basis of unified registration guidelines and strict privacy regulations. The system’s infrastructure can also be used in different contexts of public health research, including studies on the physical and psychosocial impact of pandemics as well as environmental exposures. It provides information to the stakeholders through the openly accessible Surveillance Bulletin and signals early warnings to the national and local public health authorities.

Nivel syndromic surveillance allows for the observation of patterns and trends not only among individuals who seek care, but the entire registered population. This is an important strength compared to syndromic surveillance systems that solely rely on emergency department visits [10], which provide a rapid picture of health trends but are limited to the severe cases. The monitoring of broad categories of health problems such as respiratory infections enables syndromic surveillance to serve as an early warning mechanism for novel threats, providing information earlier than laboratory confirmation [6]. The data of the Nivel syndromic surveillance system can also be used in different public health research contexts, providing insight into the extent of public health impacts, identify vulnerable populations, and guide appropriate mitigation strategies.

### Limitations

It is important to acknowledge the limitations and inherent complexities associated with syndromic surveillance. One of them is the reliance of syndromic surveillance on reporting clinical diagnoses or symptoms rather than diagnoses confirmed by a laboratory. This could introduce some degree of uncertainty, given that symptoms may not always be associated with a specific disease. In addition, it could increase the possibility of a delayed diagnosis registration compared to surveillance infrastructures that use laboratory-confirmed cases or emergency visits. Identifying rare or emerging diseases without pre-established diagnostic codes or historical data poses another challenge; a recent example is the miscoding and misclassification of COVID-19 cases in primary care, during the pandemic [30]. Establishing a reliable baseline for certain health problems can also be difficult due to different factors such as seasonality and changes in health care–seeking behavior. Technical aspects such as updates in guidelines and coding practices in primary care may also disrupt trends. Moreover, there is currently a lack of quantitative data from other surveillance systems to support a structured comparative evaluation [31] of the system’s effectiveness regarding indicators such as early detection.

### Future Directions

As the field of syndromic surveillance evolves, there are several challenges to be addressed that could substantially strengthen the utility of the existing system in public health monitoring. Despite the satisfactory national coverage rate of general practices, participation of practices in the system is voluntary and can vary per region [32]. The expansion of the system’s geographical coverage with the inclusion of more general practices across the country would strengthen the sample power as well as representativeness, allowing for a more accurate detection of disease trends and variation at the regional and population group levels. In light of climate change and emerging crises, linking syndromic surveillance with publicly available environmental exposure data could allow for the monitoring of the physical and mental impact on the population’s health more accurately, especially during seasonal events such as heatwaves or acute incidents of hazardous emissions. Another aspect would be the integration of additional data sources in the syndromic surveillance system such as those on emergency visits, similar to other established surveillance systems [9], in order to provide a more comprehensive monitoring of the population’s health. Strengthening and expanding international collaborations and networks with other syndromic surveillance systems could also help identify existing caveats and develop best practices to address them and would improve early warning of potential health threats across countries [33]. Finally, innovation should be an indispensable part of syndromic surveillance.

In particular, machine learning approaches as well as artificial intelligence are promising areas that could contribute to more accurate and efficient health monitoring, by facilitating analysis processes, improving detection algorithms, and even the development of predictive models [34].

### Conclusions

The Nivel syndromic surveillance system is an important infrastructure in providing monitoring of real world data on the occurrence of all symptoms and diseases presented in general practice, at the population level. Further implementation and refinement of syndromic surveillance methodologies towards data completeness, quality, and representativeness will enhance the system’s effectiveness in contributing to public health research and response strategies.

## Abbreviations

ADEPD: adequate record keeping in electronic patient records
ATC: anatomical therapeutic chemical
CBS: Statistics Netherlands
EHR: electronic health record
GP: general practitioner
ICPC: International Classification of Primary Care
NHG: Dutch College of General Practitioners
Nivel: Netherlands Institute for Health Services Research
Nivel-PCD: Netherlands Institute for Health Services Research-primary care database
RIVM: National Institute for Public Health and the Environment
